# Development and Validation of a 3D Resnet Model for Prediction of Lymph Node Metastasis in Head and Neck Cancer Patients

**DOI:** 10.1007/s10278-023-00938-2

**Published:** 2024-01-16

**Authors:** Yi-Hui Lin, Chieh-Ting Lin, Ya-Han Chang, Yen-Yu Lin, Jen-Jee Chen, Chun-Rong Huang, Yu-Wei Hsu, Weir-Chiang You

**Affiliations:** 1 Department of Radiation Oncology, Taichung Veterans General Hospital, Taichung City, Taiwan; 2 College of Artificial Intelligence, National Yang-Ming Chiao Tung University, Hsinchu City, Taiwan; 3 Department of Computer Science, National Yang-Ming Chiao Tung University, Hsinchu City, Taiwan; 4 Academy of Innovative Semiconductor and Sustainable Manufacturing, National Cheng Kung University, Tainan City, Taiwan; 5 Cancer Prevention and Control Center, Taichung Veterans General Hospital, Taichung City, Taiwan

**Keywords:** Head and neck cancer, Cervical lymph node metastasis, 3D Resnet model, Deep learning, Clinical decision-making

## Abstract

The accurate diagnosis and staging of lymph node metastasis (LNM) are crucial for determining the optimal treatment strategy for head and neck cancer patients. We aimed to develop a 3D Resnet model and investigate its prediction value in detecting LNM. This study enrolled 156 head and neck cancer patients and analyzed 342 lymph nodes segmented from surgical pathologic reports. The patients’ clinical and pathological data related to the primary tumor site and clinical and pathology T and N stages were collected. To predict LNM, we developed a dual-pathway 3D Resnet model incorporating two Resnet models with different depths to extract features from the input data. To assess the model’s performance, we compared its predictions with those of radiologists in a test dataset comprising 38 patients. The study found that the dimensions and volume of LNM + were significantly larger than those of LNM-. Specifically, the *Y* and *Z* dimensions showed the highest sensitivity of 84.6% and specificity of 72.2%, respectively, in predicting LNM + . The analysis of various variations of the proposed 3D Resnet model demonstrated that Dual-3D-Resnet models with a depth of 34 achieved the highest AUC values of 0.9294. In the validation test of 38 patients and 86 lymph nodes dataset, the 3D Resnet model outperformed both physical examination and radiologists in terms of sensitivity (80.8% compared to 50.0% and 91.7%, respectively), specificity(90.0% compared to 88.5% and 65.4%, respectively), and positive predictive value (77.8% compared to 66.7% and 55.0%, respectively) in detecting individual LNM + . These results suggest that the 3D Resnet model can be valuable for accurately identifying LNM + in head and neck cancer patients. A prospective trial is needed to evaluate further the role of the 3D Resnet model in determining LNM + in head and neck cancer patients and its impact on treatment strategies and patient outcomes.

**Supplementary Information:**

The online version contains supplementary material available at 10.1007/s10278-023-00938-2.

## Introduction

Accurate lymph node metastasis (LNM) assessment is essential for diagnosing and staging head and neck cancer [1, 2]. Traditional imaging diagnostic tools, including computed tomography (CT), magnetic resonance imaging (MRI), and positron emission tomography (PET), have their limitations. These diagnostic imaging tests primarily evaluate lymph nodes based on size and shape. The sensitivity of other clinical examinations, such as physical examination, or initial diagnostic strategies, such as fine-needle aspiration cytology (FNAC), for detecting LNM ranges from 60.7 to 71.4% [3–5]. The most accurate diagnosis of lymph node metastasis relies on pathological analysis. However, in clinical practice, histological screening of lymph node specimens for the presence of metastatic disease requires careful and precise execution, which is time-consuming, labor-intensive, and error-prone. As technology advances, experts have incorporated artificial intelligence (AI) into digital pathology, mainly using deep learning to solve these problems [6]. Such AI-driven techniques employ advanced algorithms to analyze pathological images, identifying intricate patterns to improve diagnosis and assist pathologists in clinical workflow [7, 8]. In addition, recent studies have applied artificial intelligence to assist LNM detection in medical imaging, and some AI models have achieved promising results [9–15].

Radiomics, a method rooted in machine learning, has been explored for its capability to distinguish lymph node metastasis (LNM) in head and neck cancer [9, 10]. However, one notable drawback of this method is the high inter-correlation among the manually crafted image features, leading to feature biases based on pre-existing assumptions [11]. Convolutional neural networks (CNNs) have the advantage of learning image features automatically, avoiding these feature biases and creating new opportunities for analysis [12]. CNNs have shown superior performance over radiomics when large datasets are available. Despite the nascent stage of employing deep learning for LNM identification, several researchers have made significant headway [13–15]. For instance, Ariji et al. utilized an 8-layer CNN to scrutinize 441 lymph nodes across 45 patients, achieving a sensitivity, specificity, and area under the receiver operating characteristic curve (AUC) of 0.75, 0.81, and 0.80, respectively [14]. Similarly, Tomita et al. designed a deep neural network for the pre-surgical diagnosis of metastasized cervical lymph nodes in oral squamous cell carcinoma (OSCC) patients using CT imaging, boasting an impressive AUC of 0.957 [15].

While CNNs have shown promise, their depth, and thus their efficacy, has been limited by the gradient vanishing problem [16]. In 2016, the Resnet network was proposed to address this problem by introducing a residual link structure and deepening the neural network from 19 layers to hundreds of layers [17]. Resnet was subsequently adapted from a 2D format to a more intricate spatial–temporal 3D variant, proving its utility on video datasets [18, 19]. In recent advancements, the 3D-Resnet has been harnessed for spatial-3D datasets, especially in medical imaging domains like MRI and CT [20–22]. In this study, we aimed to develop a deep-learning Resnet neural network to improve the precision of LNM diagnosis in patients with OSCC. Developing an accurate and efficient tool for LNM detection can potentially improve patient outcomes and reduce unnecessary treatments.

## Methods

Our investigation included patients diagnosed with head and neck cancer (HNC) who underwent neck lymph node dissection (LND) at our institute from January 2019 to March 2021. To minimize the impact of preoperative CT scans on diagnostic accuracy for cervical nodal metastases, we selected patients who had received a diagnostic intravenous contrast-enhanced CT scan of the neck within 30 days before LND. We gathered demographic, clinical, and pathological data, which featured the anatomical site, tissue dimensions, microscopic characteristics, and lymph node assessment for malignant cells. We collected paired preoperative contrast-enhanced CT scans from 156 individual HNC patients with corresponding pathological reports. These scans were conducted using three distinct CT scanners from two leading manufacturers: Revolution CT (GE Healthcare), Brilliance 64 (Philips Healthcare), and iCT 256 (Philips Healthcare). In adherence to the Helsinki Declaration’s ethical guidelines, our study received approval from the Institutional Review Board.

## Establishing Ground Truth

The cervical lymph node annotation was manually contoured slice-by-slice in the axial plane for each CT scan, and the segmentations were labeled as either “lymph node metastasis negative (LNM-)” or “lymph node metastasis positive (LNM +)” based on the corresponding LND pathology report. To ensure accuracy, the dissected lymph nodes were tagged according to laterality, neck level, surrounding tissue type, and nodal size, as determined from a correlative evaluation of the pathology report (Supplementary 1). The segmentations and labels were saved as Radiotherapy Structure Set (RTSS) files using Varian v15.1 radiation planning software. Two radiation oncologists reviewed the segmentation accuracy to ensure consistency in establishing the ground truth for lymph node metastasis. These nodes were carefully examined and confirmed pathologically by radiation oncologists. Following this, they were labeled and captured in computed tomography images. The harvested segmentations and labels formed the foundational dataset for training and testing the 3D-Resnet neural network.

## Image Processing and Deep Learning Model

In the preprocessing image phase, a 3D binary mask for each lymph node was produced. The lymph node HU values were cropped according to the boundary of the contours from RTSS contours, resulting in a 3D image of the lymph node called “size-preserved” lymph node imaging, which was placed in the center of a bounding box backgrounded by zeroed voxels. Another type of region of interest (ROI) was generated from the “size-preserved” image, called “size-invariant” ROIs, which were created to compare the effect of different input information. The study used a ratio of 3:1 to randomly split the training and test datasets from the lymph nodes, with LNM-negative data duplicated to balance the two labels to a 1:1 ratio. Augmentation skills of rotation and flip were applied to increase the training data numbers to 16-fold.

**Fig. 1:**
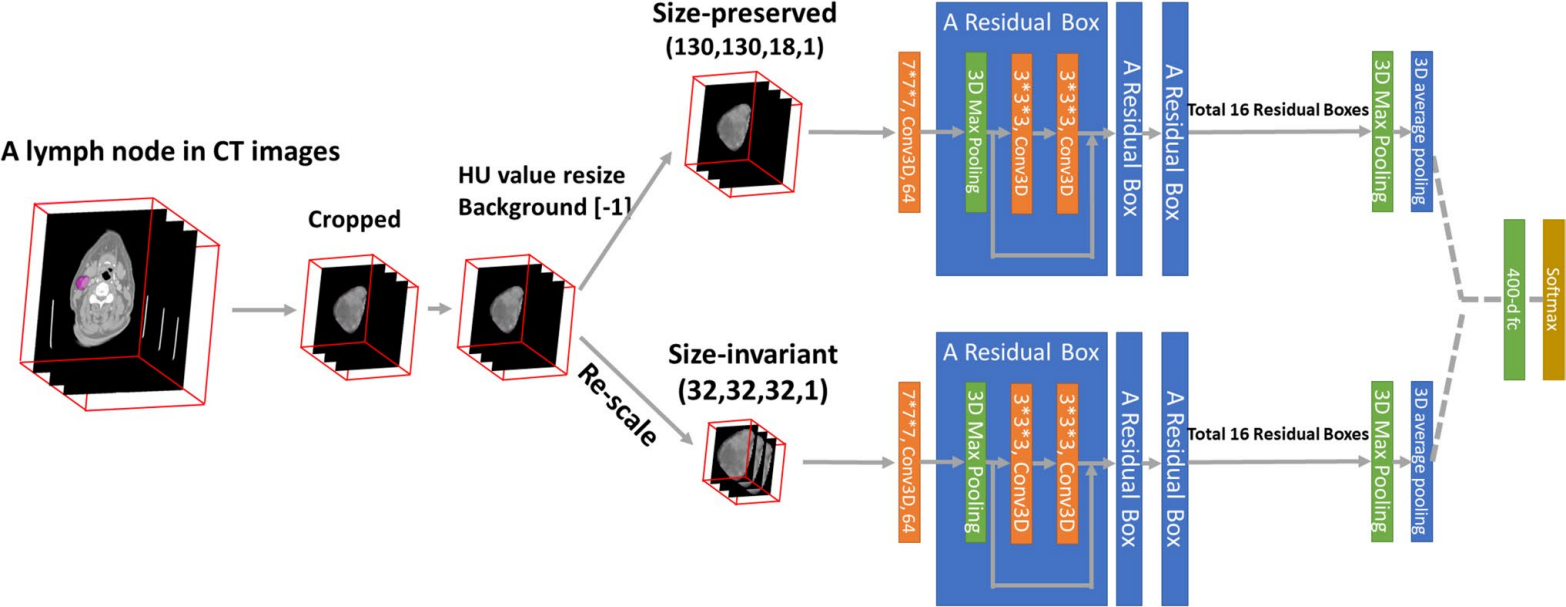
Dual-pathway 3D-ResNet architecture featuring both size-preserved and size-invariant pathways

The study proposed a dual-pathway 3D-CNN network with residual connections (3D-Resnet), with the two pathways designed to receive input from the size-preserved and size-invariant images. Both pathways were constructed with 34 convolutional layers and joined after feature extraction to predict a dual-label output of either LNM-positive or LNM-negative. The architecture of the 3D-Resnet model is displayed in Fig. 1. To prevent overfitting, batch normalization layers, dropout layers, and L2 regularization were utilized, with the output probabilities obtained by applying sigmoid classifiers. The study performed ablation experiments to optimize the architecture of the proposed model, testing single pathway models in different depths and exploring the best depth of dual-3D-Resnet by trying different depths of convolutional layers and recording their performance. The Adam optimizer tuned the network weights during the training phase with a mini-batch size of 16 and an initial learning rate of 0.001. The selected models underwent four-fold cross-validation to ensure robustness. The study used Python v3.9.12, Pytorch v1.9, SciPy 1.8.0, sci-kit-learn v1.0.2, sci-kit-image v0.19.2, and SimpleITK v2.2 packages to create and implement the network. The algorithm’s training ground was an RTX 2080 Ti graphics processor unit, and the proposed code is publicly available on GitHub (https://github.com/acqxi/hnc).

## Evaluation Metrics Overview

The evaluation metrics employed in this study encompassed accuracy, sensitivity, specificity, positive predictive value (PPV), negative predictive value (NPV), and area under the receiver operating characteristic curve (AUC-ROC) for both training and test sets. An algorithm was employed to measure lymph node dimensions and volume to minimize interobserver bias. Lymph nodes were manually contoured slice-by-slice in the axial plane, and each segmentation was classified as LNM- or LNM + based on the corresponding LND pathology report. Lymph node volumes were calculated using the ellipsoid formula, considering the *X*, *Y*, and *Z* dimensions in pixels and slice thickness [23]. Accuracy refers to the proportion of accurately classified samples relative to the total number, while sensitivity measures the correct identification rate of LNM + cases by the model. AUC-ROC serves as a comprehensive metric for evaluating the model’s capacity to differentiate between LNM + and LNM- cases, with a value of 1 signifying perfect discrimination and 0.5 indicating no discrimination. Data were entered and analyzed in Microsoft Excel for percentage calculation. Statistical analysis considered sensitivity, specificity, PPV, NPV, and Kappa coefficient were calculated using SPSS software version 23.

## Results

Table 1 lists the clinical and pathological data on the primary tumor site and clinical and pathological T and N stages of the 156 patients in this study. Within this cohort, each patient exhibited a range of 1 to 8 lymph nodes, culminating in 341 lymph nodes. The analysis of labeled-extracted lymph nodes revealed that LNM + lymph nodes were significantly larger across the *X*, *Y*, and *Z* dimensions (*p* < 0.001), as well as in volume (*p* < 0.001) when compared to LNM- lymph nodes (Table 2). These findings align with previous studies concerning lymph node metastasis in head and neck cancer, corroborating the clinical consensus. Subsequent ROC curve analysis was conducted to ascertain the optimal cutoff point for predicting LNM + , yielding 12.8 mm, 11.2 mm, 14.0 mm, and 15.5 ml for *X*, *Y*, and *Z* dimensions and volumes, respectively. Interestingly, the sensitivity and specificity values demonstrated variability based on size or volume, wherein the *Y* and *Z* dimensions exhibited the highest sensitivity (84.62%) and specificity (72.15%), respectively.

**Table 1:** Patients’ demographic data and TNM stage

Patient characteristics				*n*	%
Sex	All			156	100%
	Male			141	90.4%
	Female			15	9.6%
Age	Medium (range)			58 (34–85)	
Primary cancer				*n*	%
Lip				6	3.8%
Tongue				51	32.7%
Alveolar ridge				25	16.0%
Floor of mouth				8	5.1%
Palate				8	5.1%
Buccal				44	28.2%
Oropharynx and tonsil				12	7.7%
	p16( +)			8	5.1%
	p16(-)			4	2.6%
Pyriform sinus				1	0.6%
Unknown primary				1	0.6%
Clinical T	*n*	%	Pathology T	*n*	%
T0	1	0.6%	T0	3	1.9%
Tis	0		Tis	2	1.3%
T1	37	23.7%	T1	42	26.9%
T2	54	34.6%	T2	47	30.1%
T3	14	9.0%	T3	26	16.7%
T4	1	0.6%	T4	0	0.0%
T4a	44	28.2%	T4a	35	22.4%
T4b	5	3.2%	T4b	1	0.6%
Clinical N	*n*	%	Pathology N	*n*	%
N0	88	56.4%	N0	100	64.1%
N1	29	18.6%	N1	18	11.5%
N2	1	0.6%	N2	1	0.6%
N2a	4	2.6%	N2a	7	4.5%
N2b	21	13.5%	N2b	11	7.1%
N2c	11	7.1%	N2c	3	1.9%
N3	1	0.6%	N3	0	0.0%
N3b	1	0.6%	N3b	16	10.3%
Clinical stage	*n*	%	Pathology stage	*n*	%
0	2	1.3%	0	4	2.6%
1	30	19.2%	1	39	25.0%
2	31	19.9%	2	29	18.6%
3	25	16.0%	3	25	16.0%
4A	62	39.7%	4A	42	26.9%
4B	6	3.8%	4B	17	10.9%

**Table 2:** The dimensions of lymph nodes and its prediction values

Dimensions and volume					
LNM	*n*	*X*-axis	*Y*-axis	*Z*-axis	Volume
Positive	104	17.53 ± 7.71	17.42 ± 7.07	19.19 ± 7.86	4.52 ± 3.68
Negative	237	11.85 ± 3.53	10.95 ± 3.02	12.03 ± 5.34	0.96 ± 0.87
*P* value		< 0.001**	< 0.001**	< 0.001**	< 0.001**
ROC curve for LNM					
AUC		0.803	0.737	0.776	0.793
Cuff value		12.8	11.2	14.0	15.5
*P* value		< 0.001	< 0.001	< 0.001	< 0.001
Prediction value for LNM					
Sensitivity		69.2%	84.6%	64.4%	77.9%
Specificity		68.8%	60.3%	72.2%	64.6%
PPV		68.9%	67.9%	N/A	68.6%
NPV		69.1%	79.8%	N/A	74.6%

Chi-square test or independent *t*-test

*LNM* lymph node metastasis, *PPV* positive predictive value, *NPV* negative predictive value, *ROC* receiver operating characteristic curve, *AUC* area under the ROC curve

*p < 0.05; **p < 0.01

Table 3 delineates the variants of 3D-Resnet models explored in this study, including the Single-3D-Resnet and Dual-3D-Resnet, along with input size and model depth alterations. It documents the AUC value, the number of model parameters, memory usage, and GFLOPs for each model variant. The findings reveal that the Dual-3D-Resnet model, at a depth of 34 and input sizes of 32 × 32 × 32 or 130 × 130 × 18, exhibits the highest AUC values of 0.929 and 0.914, respectively, outperforming the Single-3D-Resnet model (Supplementary 2).

**Table 3:** 3D Resnet model performance

	**Single-3D-Resnet**				**Dual-3D-Resnet**			
Input size	130 × 130 × 18		32 × 32 × 32		Both		Both	
Model	1	2	3	4	5	6	7	8
Model depth	10	18	34	10	18	34	34/18	34/34
Params (MB)	55	126	242	55	127	242	369	484
Memory (MB)	224	344	528	72	149	271	676	798
GFLOPs	11.2	20.6	37.1	1.0	1.8	3.2	19.5	40.2
ROC curve analysis								
AUC	0.906	0.909	0.897	0.883	0.894	0.890	0.914	0.929
Cuff value	0.6867	0.5425	0.6261	0.6596	0.7993	0.4371	0.5221	0.1365
* P* value	< 0.001	< 0.001	< 0.001	< 0.001	< 0.001	< 0.001	< 0.001	< 0.001

*ROC* receiver operating characteristic curve, *AUC* area under the ROC curve

**Fig. 2:**
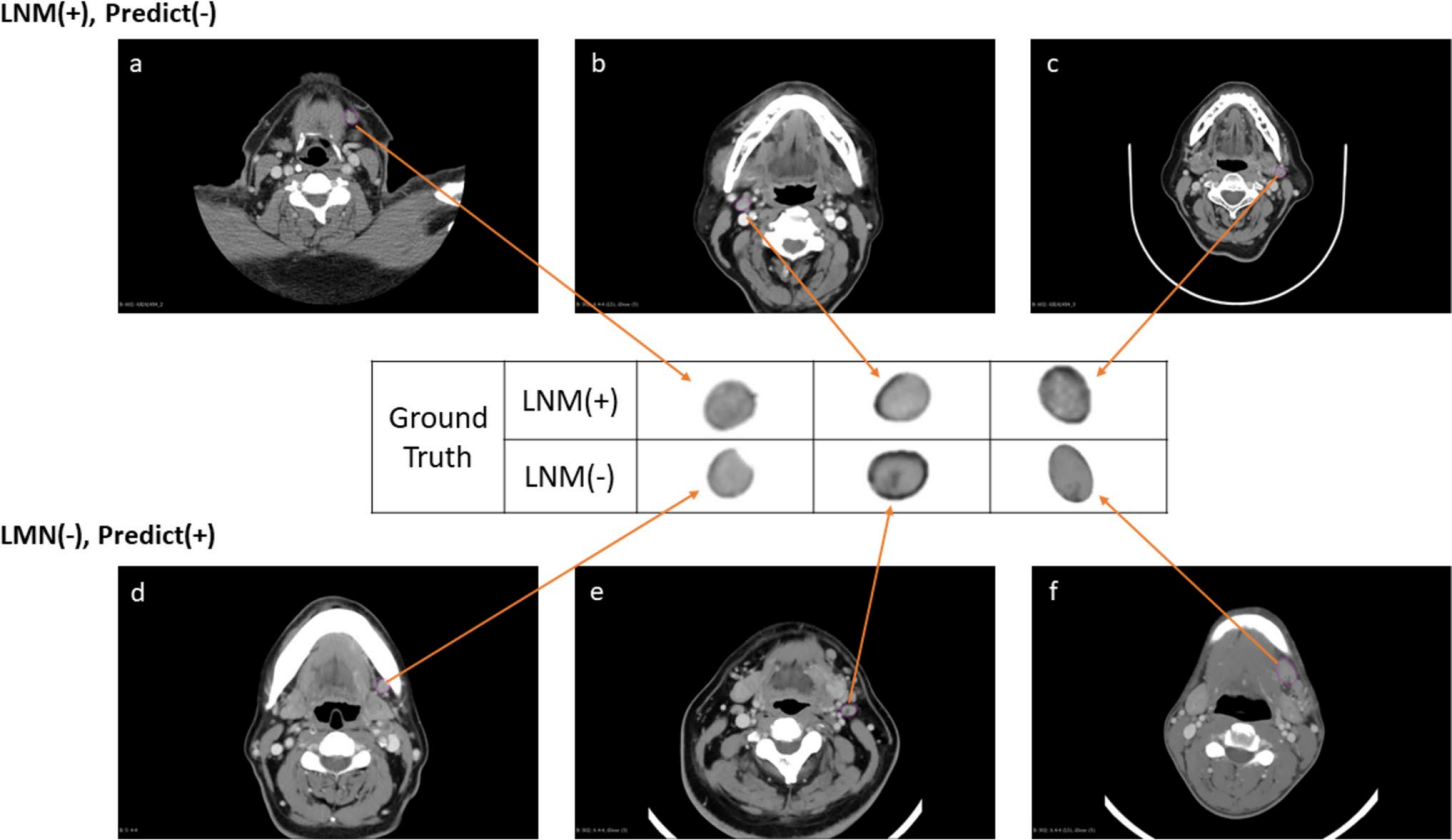
Illustration of cases from 3D ResNet predictions: (a–c) showcase LNM + cases with incorrect predictions alongside their corresponding CT scan features; (d–f) display LNM- cases with inaccurate predictions and their respective CT scan features

We expanded our examination of the efficacy of the 3D Resnet model in predicting LNM + by analyzing 86 lymph nodes in 38 patients with head and neck cancer (Fig. 2). We further compared these results with the clinicians’ physical examinations and the radiologists’ assessments based on CT scans (Table 4). The 3D Resnet model exhibits a high specificity of 90.0% and a sensitivity of 80.8%, showing a solid ability to identify positive cases. Moreover, the 3D Resnet model achieves the highest PPV of 77.8% and NPV of 91.5% among all methods in the lymph node analysis, indicating its proficient accuracy in predicting both positive and negative cases. The Kappa statistic of 0.700 further underscores the substantial agreement between the predicted and actual values by the 3D Resnet model, which is notably higher than other methods in patient and lymph node analysis contexts.

**Table 4:** Comparison of the perdition values for LNMs between different methods

Prediction values	By patient			By lymph node
	Clinician PE	Radiologists	3D Resnet	3D Resnet
Sensitivity	50.0%	91.7%	75.0%	80.8%
Specificity	88.5%	65.4%	80.8%	90.0%
PPV	66.7%	55.0%	64.3%	77.8%
NPV	79.3%	94.4%	87.5%	91.5%
Kappa	0.412	0.443	0.534	0.700
*P* value	0.01*	0.002**	0.001**	< 0.001**

Kappa test

*PE* physical examination, *LNM* lymph node metastasis, *PPV* positive predictive value, *NPV* negative predictive value

^*^p < 0.05; **p < 0.01

## Discussion

The findings from our study underscore the limitations inherent in the current preoperative diagnostic tools, including physical examination and radiological diagnosis, for accurate staging and devising treatment strategies for patients with head and neck cancer. This notion is further corroborated by the modest concordance between clinical and pathological N-stage classification observed among the 156 patients evaluated in this study, reflecting a need for more reliable preoperative diagnostic methodologies as echoed in prior literature [24]. Lymph node size has been identified as an essential factor in predicting lymph node metastasis, and our study confirms this finding [25, 26]. However, it is imperative to integrate lymph node size assessment with other clinical and radiographic indicators such as location, morphology, and presence of necrosis. As demonstrated in our study, experienced radiologists can attain high diagnostic performance by synthesizing these imaging findings, achieving a sensitivity of 91.7% and a specificity of 65.4%. Nonetheless, novice practitioners might not reach similar levels of diagnostic proficiency. Our study also suggests that advanced deep learning techniques like 3D Resnet can further strengthen the predictive values of these tools.

In addressing the challenges of accurate diagnosis, it is noteworthy to consider the performance of existing imaging modalities. The diagnostic accuracy of computed tomography (CT) scans in identifying positive cervical lymph nodes can be influenced by various factors [27, 28]. Within this scope, multiple studies have shown that PET-CT exhibits higher sensitivity and specificity compared to MRI and CT [29, 30]. Depending on the maximum standardized uptake value (SUV-max) threshold and cancer type, the sensitivity of PET-CT in detecting LNM + ranges between 75 and 84%, the specificity between 59.4 and 87%, the PPV between 19.1 and 75%, and the NPV between 94 and 95.7% [31, 32]. However, a limitation associated with employing PET-CT for detecting cervical lymph node metastasis in head and neck cancer is the potential for false-positive or false-negative results, which could lead to unnecessary surgical interventions and increased radiation exposure. Our study provides evidence that the 3D Resnet model has the potential to accurately identify LNM + and provide valuable information in determining surgical candidates, identifying surgical areas, and performing pixel-level risk analysis to minimize radiation exposure while preserving patient quality of life.

The success of radiotherapy is highly dependent on the accuracy of target volume delineation, and CT simulation is an integral component of the planning process [33, 34]. However, determining the appropriate irradiation of the neck lymph node area can be challenging in head and neck cancer due to the complex anatomy and variability of lymph node involvement [35]. Incorrect delineation can result in under- or over-treatment, compromising treatment efficacy and increased toxicity [36]. The decision to irradiate the neck lymph node area is influenced by factors such as the primary tumor’s location and extent, the risk of lymph node involvement based on clinical and pathological data, and the individual patient’s risk factors and treatment goals [2, 37, 38]. The findings of this study suggest that the 3D Resnet model could assist radiation oncologists in accurately delineating the lymph node region and identifying patients at high risk of LNM + . With the advancements in CT simulation technology, advanced radiation therapy can achieve a more conformal dose distribution, improving tumor control rates and reducing treatment-related toxicity.

Despite our study’s promising results, several limitations must be considered. Firstly, the study was conducted using data from only one medical center, which may limit the generalizability of the findings. Further external validation using data from multiple centers or a federated learning framework is necessary to ensure the model’s robustness and generalizability. Secondly, a lymph node detection and auto-segmentation model is required before implementing the recognition model into the clinical workflow. Lastly, we did not integrate the primary tumor area imaging data and clinical features, such as tumor biomarkers with lymph node ROI, to build a more robust model for LNM prediction. A further study could provide more robust evidence on the performance of the 3D Resnet model and its potential impact on patient outcomes.

### Supplementary Information

Below is the link to the electronic supplementary material.

Supplementary file1 (DOCX 1021 KB)

## Data Availability

The datasets generated and/or analyzed during the current study are not publicly available due to privacy and ethical considerations. However, they are available from the corresponding author upon reasonable request and with permission of the institutional review board and compliance with relevant data protection regulations. This research complies with all relevant ethical regulations. All patient-related data was de-identified to protect privacy and confidentiality. For access to the data, researchers must adhere to the guidelines stipulated by the institutional review board and ensure that their research objectives align with the ethical standards and purposes for which the data was originally collected.
