# Supplementary material for: Development and Validation of a 3D Resnet Model for Prediction of Lymph Node Metastasis in Head and Neck Cancer Patients
Source: J Imaging Inform Med. 2024 Jan 16;37(2):679–87. doi: 10.1007/s10278-023-00938-2 (PMC11031546; doi:10.1007/s10278-023-00938-2)

Supplementary 1. Methodology for Correlative Evaluation between Pathological Reports and CT Scan Image Reports in Lymph Node Metastasis Assessment

In the scope of our investigation, a thorough examination of metastatic lymph nodes in subjects diagnosed with head and neck cancer was undertaken. Herein, we delineate the methodologies employed and the subsequent findings:

1. Lymph Node Assessment:

Most of the patients in our study exhibited 1 to 2 metastatic lymph nodes within each delineated neck-level region.

1. Identification Techniques:

A comprehensive approach was employed to accurately identify the specific lymph nodes. This involved:

1. Utilizing surrounding anatomical structures as landmarks. For instance, the submandibular gland was used as a reference point (illustrated within a green box in the accompanying diagrams).
2. Noting marked size distinctions of the lymph nodes (highlighted within a red box in the accompanying diagrams). An exemplary lymph node was identified with a dimension of 3.5 cm.
3. Subject Exclusion:

Five patients were excluded from the study following the lymph node identification process. The exclusions were necessitated due to challenges in lymph node identification from the pathological reports.

| 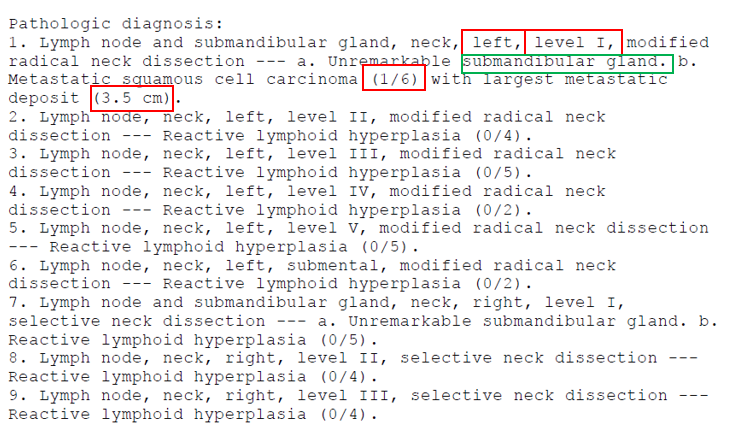 |
| --- |
| 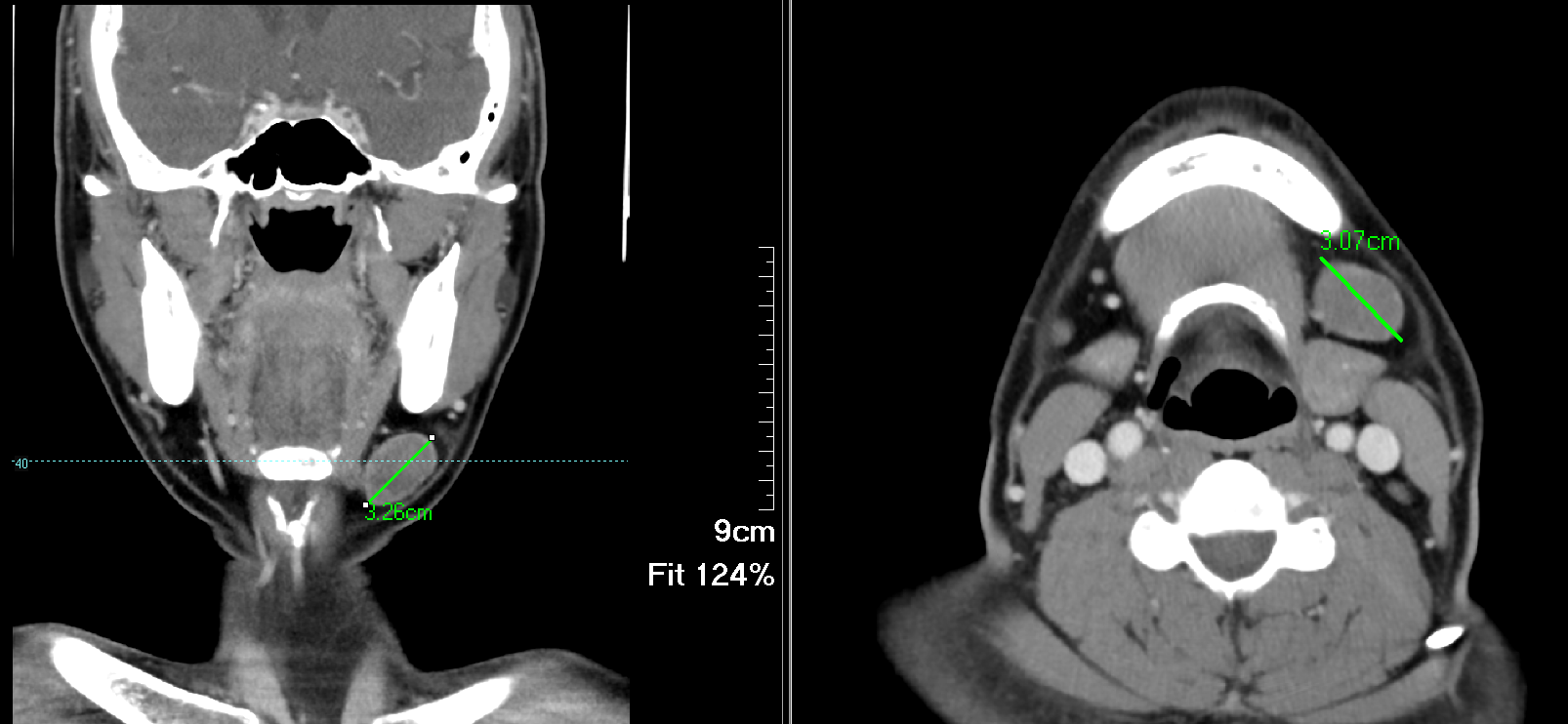 |
| *We utilize the laterality, neck level, diameter, and surrounding tissue to identify the forementioned lymph node(s). |

Supplementary 2. Performance Analysis of Different 3D ResNet Models in Lymph Node Metastasis Detection


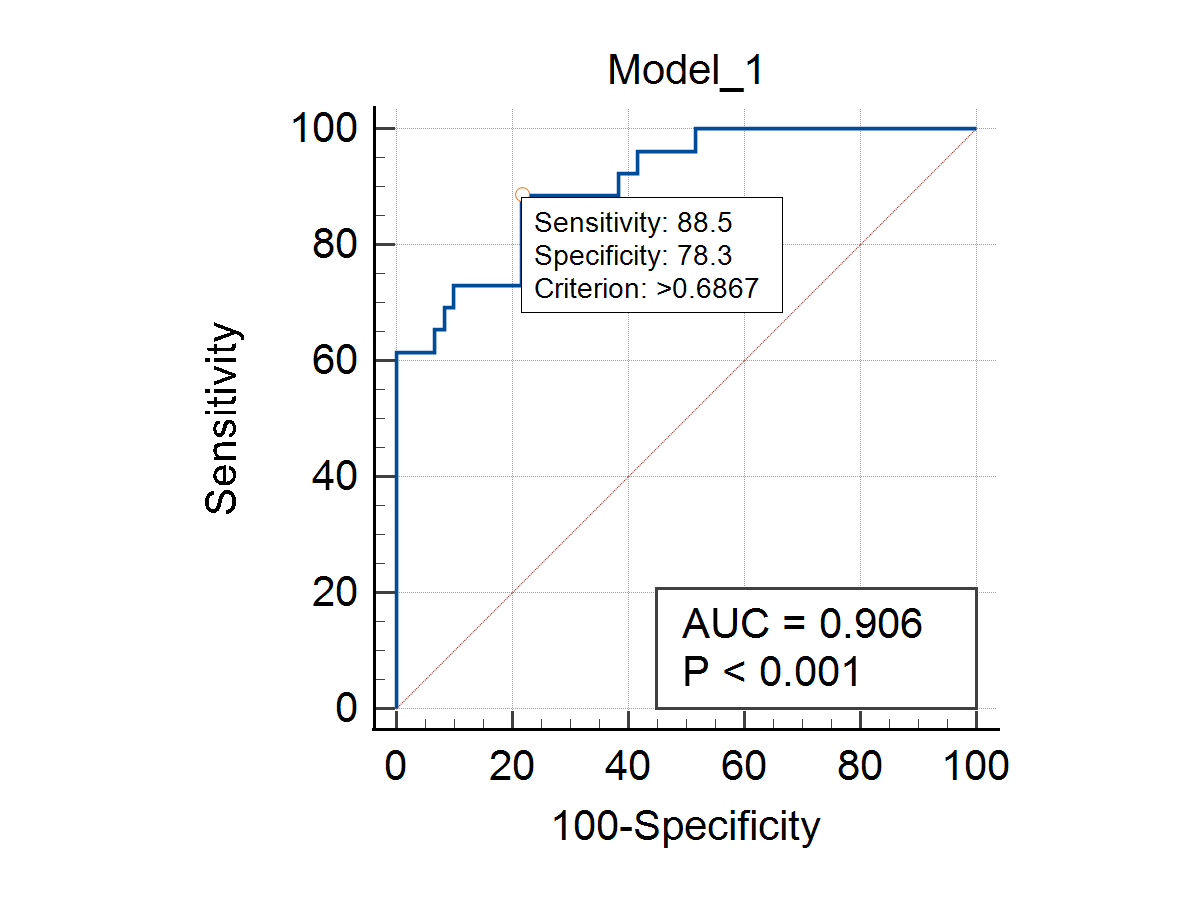

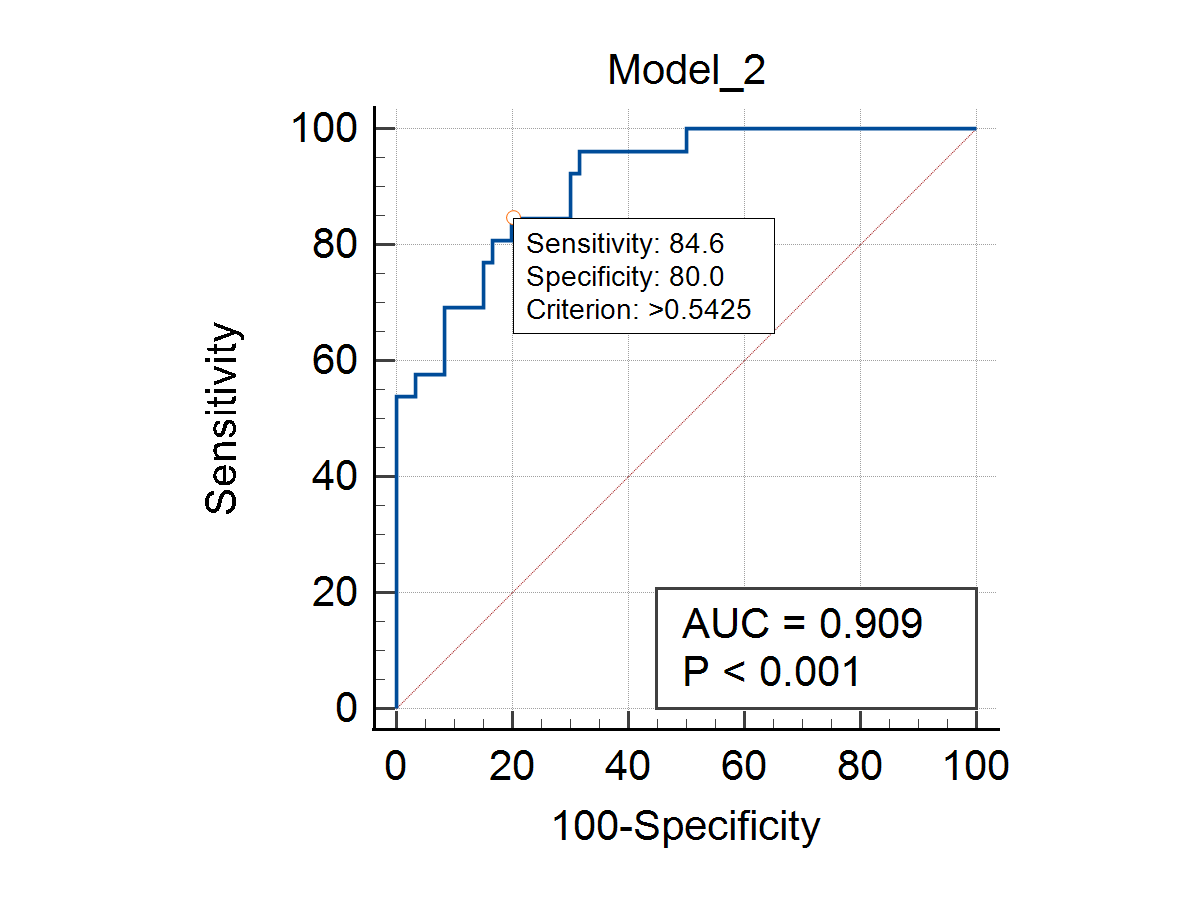

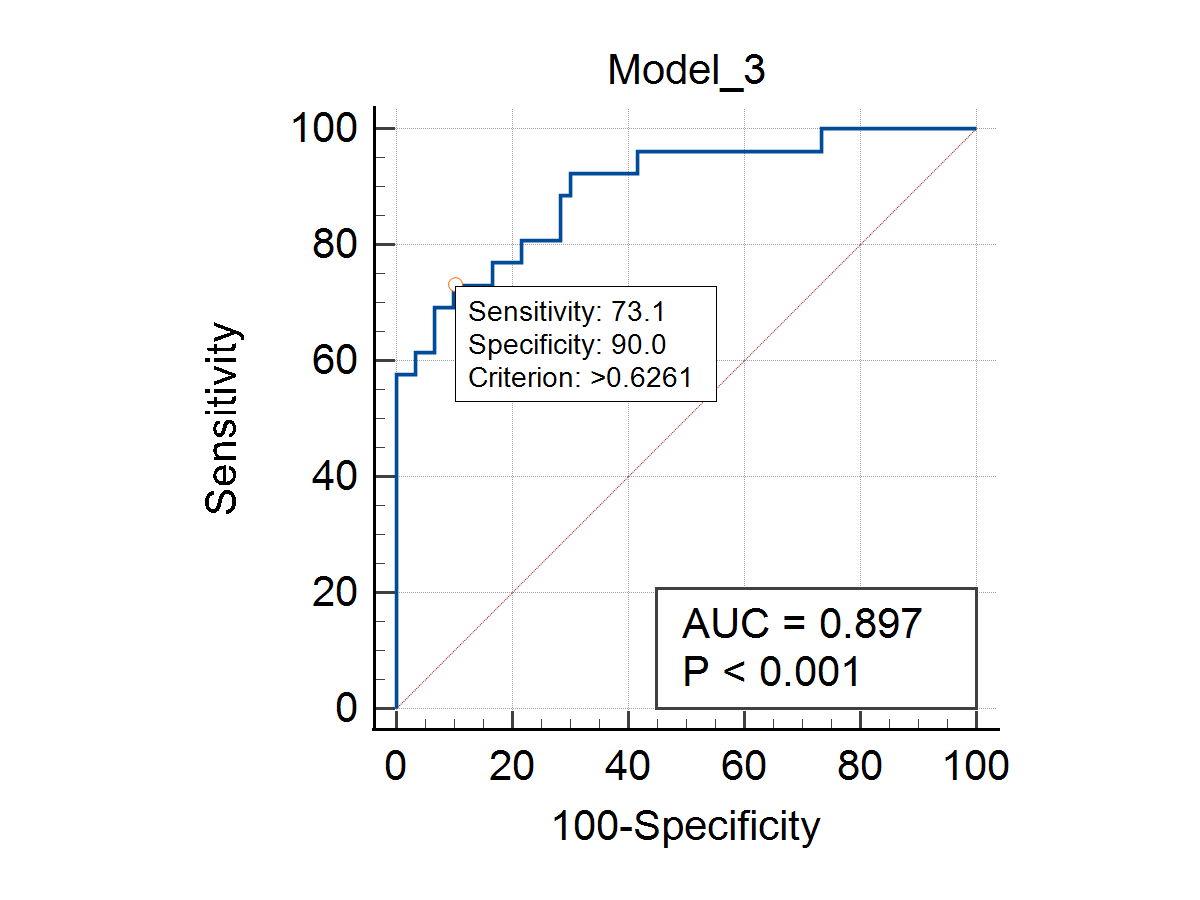

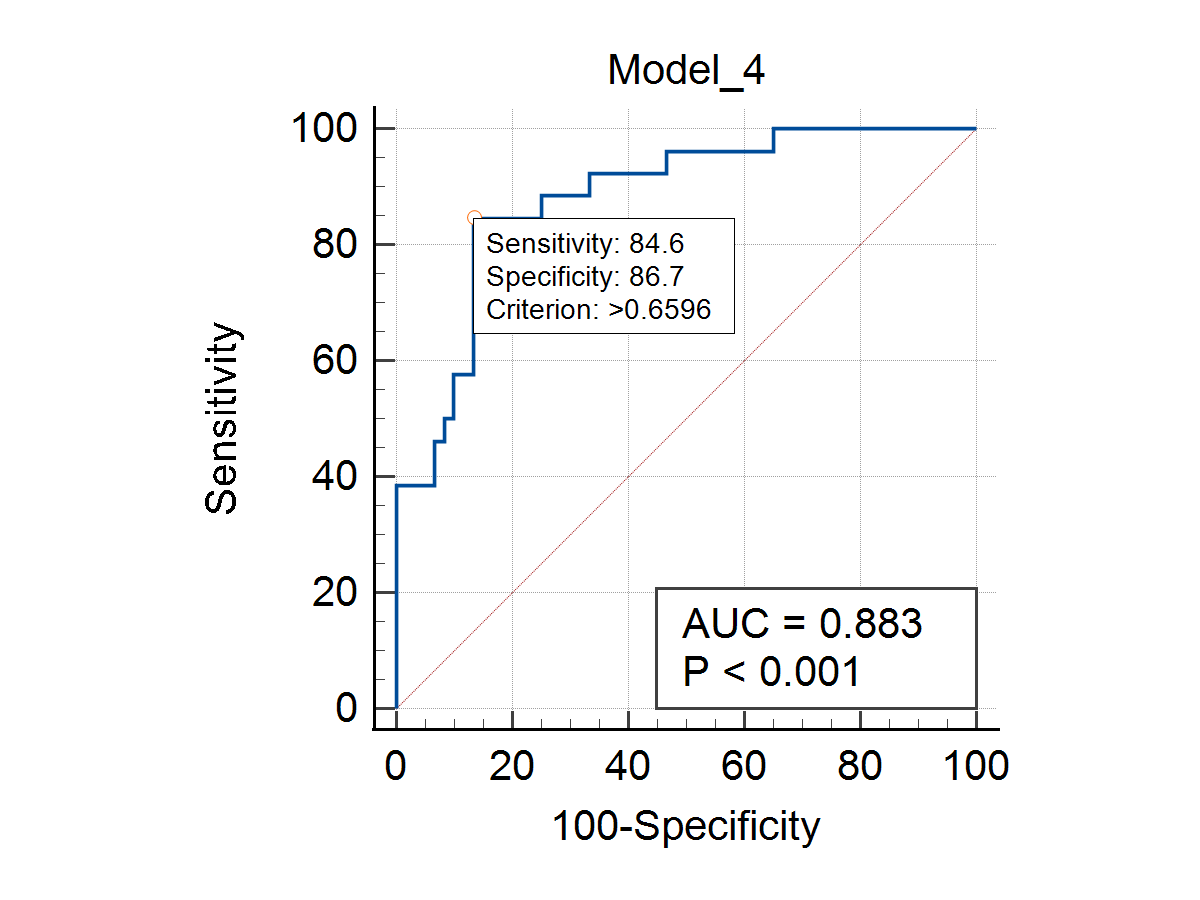

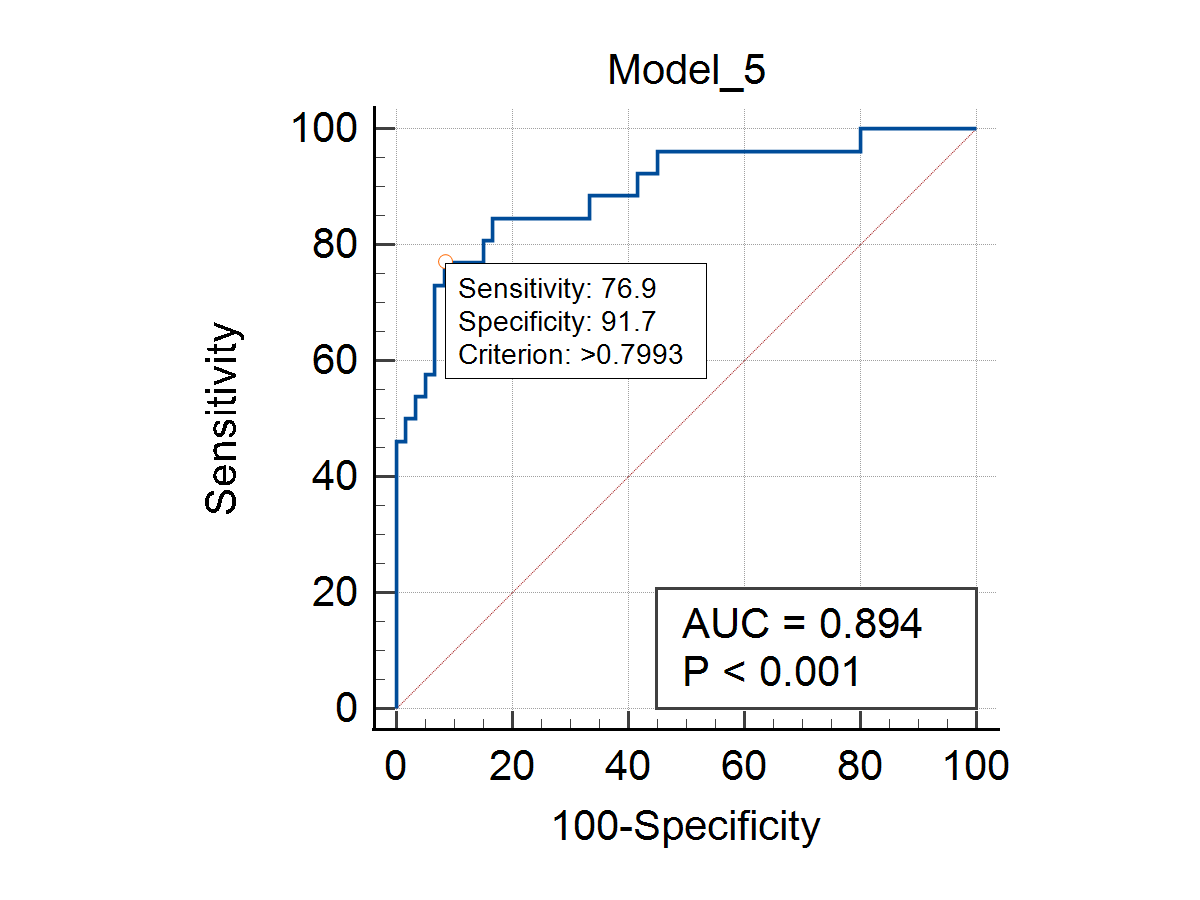

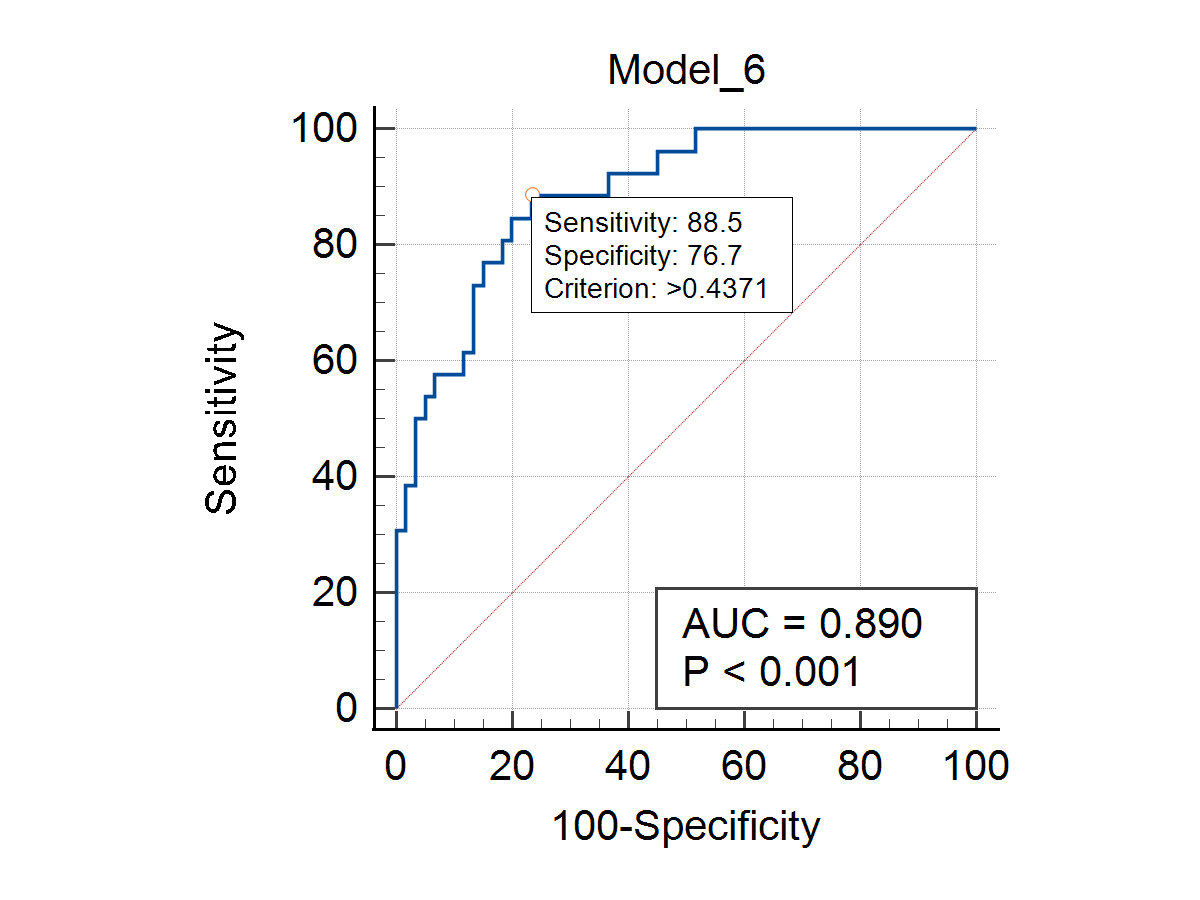

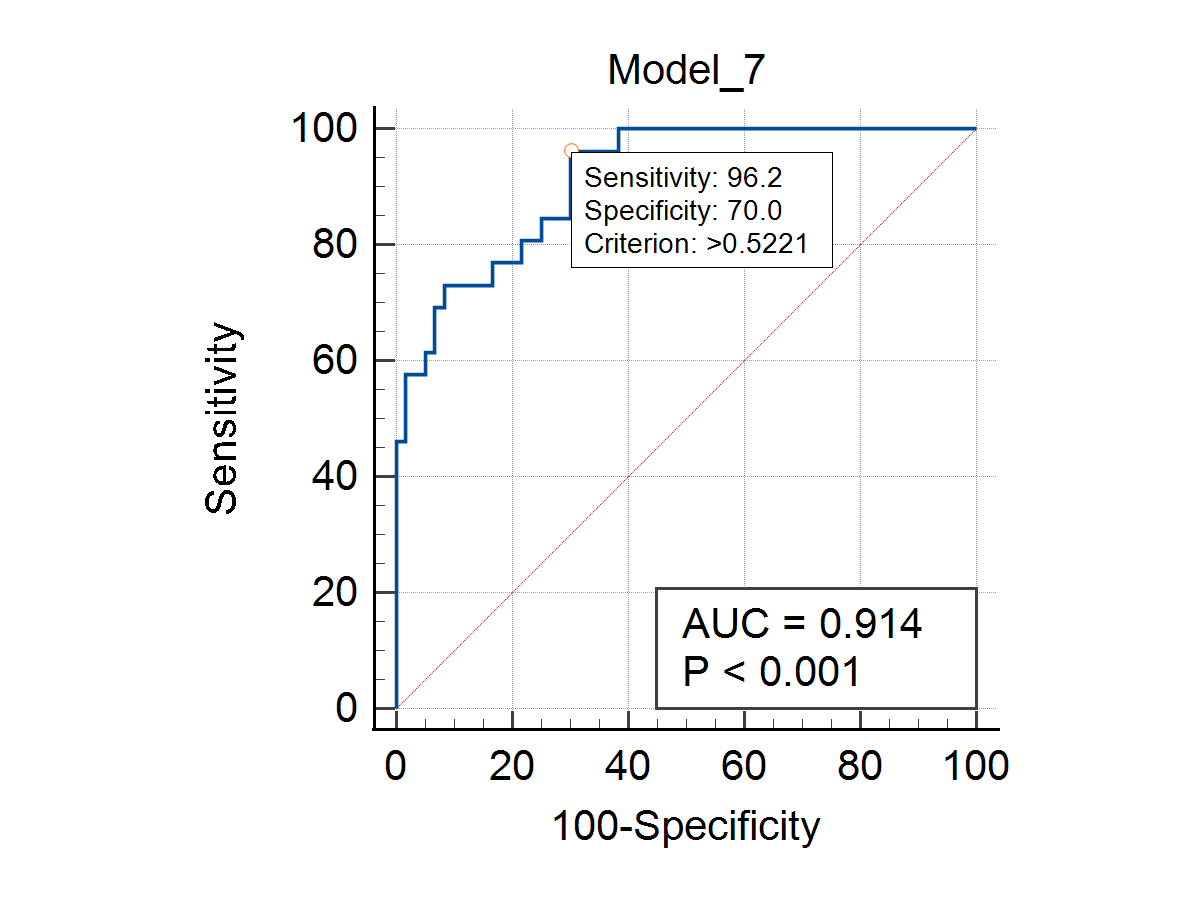

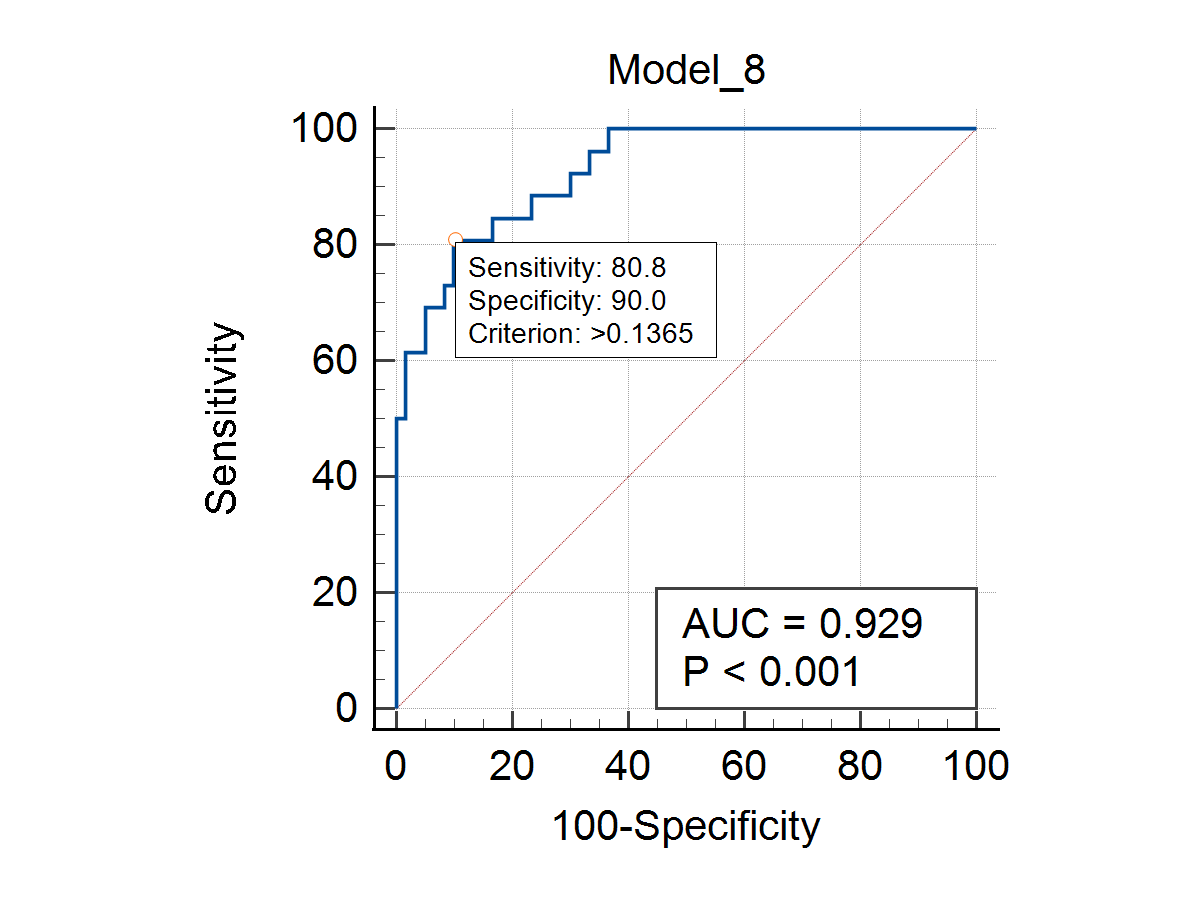

Supplement: Supplementary file 1 — Supplementary file1 (DOCX 1021 KB) [file 10278_2023_938_MOESM1_ESM.docx]
